# The Brazilian comprehensive response to hepatitis C: from strategic thinking to access to interferon-free therapy

**DOI:** 10.1186/s12889-016-3784-4

**Published:** 2016-11-02

**Authors:** Fabio Mesquita, Melina Erica Santos, Adele Benzaken, Renato Girade Corrêa, Elisa Cattapan, Leandro Soares Sereno, Marcelo Contardo Moscoso Naveira

**Affiliations:** 1 Department of STI, AIDS and Viral Hepatitis, Secretariat of Health Surveillance, Ministry of Health, Setor Administrativo Federal Sul 02-Bloco F-Ed. Premium Torre I, Brasília, 70070-600 Federal District Brazil; 2 Pan American Health Organization, World Health Organization, Avenida das Nações-Setor de Embaixadas Norte, Lote 19, Brasília, 70312-970 Federal District Brazil

**Keywords:** Hepatitis C, Health access, Interferon-free

## Abstract

**Background:**

Hepatitis C affects over 185 million people around the world. This silent disease is responsible for up to 700,000 deaths per year. Despite the scientific revolution in diagnosis and treatment, hepatitis C control remains a huge challenge due to the cost of effective medications.

In response to the global outcry of hepatitis epidemic and the need to improve the nation’s public health response, the Ministry of Health of Brazil revolutionized hepatitis C treatment by incorporating highly effective drugs that can be accessed through sustainable and universal means.

**Discussion:**

This paper describes the unique process of implementing evidence-informed policy to respond to hepatitis C epidemic through the update of hepatitis C treatment in Brazil based on the estimate of disease prevalence, current international guidelines, and the cost-effectiveness impact in the Brazilian Unified Health System. Through a debate of an experience report, the authors underlie the strategic plan implemented according to the situation analysis that emphasized the need to improve its current response over a relatively short-term period. The comprehensive response is detailed comprising three main objectives: improve treatment outcomes by evaluating and incorporating new and effective medications at a sustainable price; elaborate on clinical guidelines to treat hepatitis C patients; and develop awareness and diagnosis campaigns targeted at the population of interest. In this scenario, Brazil was able to obtain an unprecedented discount for a high-medium income country; provided treatment to more than 7000 individuals in the last 2 months of 2015; and expects to treat 38,000 new patients in 2016.

**Summary:**

The remarkable process applied in Brazil was developed according to epidemiological data and scientific evidence, and it was motivated by the engagement of the country in the Sustainable Development Goals, which may inspire other developing countries to identify ways to achieve these goals by 2030.

## Background

Over 185 million people are infected with the hepatitis C virus (HCV) worldwide [1, 2]. HCV infection is usually asymptomatic, with symptoms occurring mostly at advanced stages of the disease. Its late diagnosis contributes to transmission of the disease and its associated high mortality. It is estimated that 350,000 to 700,000 deaths could occur annually exclusively due to HCV infection [1, 3].

There were no means of diagnosing HCV infection until early 90’s, when tests finally became available and routinely performed in blood banks [4]. Such restriction of technology in diagnosis was also common to therapeutics, which until recently was mostly acknowledged by poor treatment adherence and efficacy and high rates of adverse events.

Recent scientific developments have granted health professionals and patients a new opportunity to defeat hepatitis C [5, 6]. Improved understanding of the virus, as well as the research and development of new medications, has resulted in new and effective treatments and a promising pipeline for years to come [7].

Unfortunately, such success is reserved for those who can afford expensive new treatments and is beyond the reach of developing countries, which constitute the majority of the world’s population. The global scientific community and civil society have objected to the financial restrictions imposed on the right to health [8].

To improve the nation’s public health response, the Ministry of Health (MoH) of Brazil decided to act and to revolutionize hepatitis C treatment by incorporating highly effective drugs with sustainable and universal access, according to the principles of the country’s Unified Health System (SUS)–universality, integrality and equity. The division responsible for this feat was the Department of STI, AIDS and Viral Hepatitis (DDAHV). From the outset, the Health Minister’s Office, the Secretariat of Health Surveillance (SVS), the Secretariat of Science, Technology and Strategic Inputs (SCTIE), the Brazilian Health Surveillance Agency (ANVISA), organized civil society and the scientific community, supported the initiative. This joint effort resulted in significant positive changes to public health policy in Brazil.

Different countries need specific strategies to address viral hepatitis, given the variability in epidemiology and public health infrastructure. However, we understand that many countries willing to fight to control hepatitis C, as a public health problem (according to the Sustainable Development Goals), would benefit from the strategic thinking applied by the MoH of Brazil during this next phase of hepatitis C assistance.

This paper aims to describe the unique process of implementing evidence-informed policy to respond to hepatitis C epidemic through the update of hepatitis C treatment in Brazil including an interferon-free therapy, with the direct-acting antiviral treatment, based on the up-to-date estimate of disease prevalence in the country, current international guidelines, and cost-effectiveness impact in SUS.

## Discussion

At first, the MoH of Brazil in partnership with the University of Sao Paulo Medical School developed a mathematical model to estimate the number of people living with chronic hepatitis C [9]. The mathematical model was performed using figures from the most recent 12 years from two major national databases: the Information System on Diseases of Compulsory Declaration (SINAN) and the Brazilian System of Transplants (SNT/MS) [10].

**Fig. 1:**
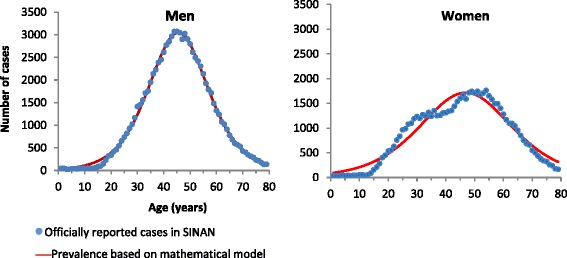
Hepatitis C cases according to age and gender, Brazil, 2008–2014

The model estimated that 1.4 to 1.7 million people are chronically infected with HCV in Brazil. Recent figures from the national datasets (2008–2014) elucidated that the population aged 40 and older is the group most affected by hepatitis C. These people were most likely infected before 1993, when blood safety was not optimal and injection drug use was still a public health issue, and they were diagnosed 15 to 30 years later (Fig. 1) [11, 12].

Further development of the mathematical model, it was estimated that to control hepatitis C the country would not only need to increase the treatment effectiveness from 50 to 90 % chance of cure but also make considerable efforts to expand treatment numbers, increasing from an average of 15,000 treatments to 45,000 treatments per year [9, 11]. Such findings have raised concerns about the extent of investment required to control hepatitis C, and provided insight regarding the ongoing progression of undetected disease, the likelihood of accumulated comorbidities, and the development of cirrhosis and hepatocellular carcinoma—greatly affecting the health system in the future.

**Fig. 2:**
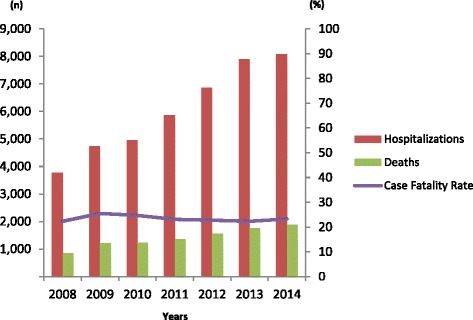
Hospitalizations, deaths and case fatality rates due to liver cancer, Brazil, 2008–2014

So next, the DDAHV assessed care and treatment individual registers through national databases of hospitalizations due to hepatocellular carcinoma, viral hepatitis and liver transplants. Despite modern advances in more complex medical care and significant improvements in waiting lists and liver transplant numbers, over the last 7 years, hospitalizations due to hepatocellular carcinoma increased greatly, reaching an average case fatality rate of approximately 23 %–probably as a result of the high prevalence of chronic hepatitis C (Fig. 2).

Additionally, the DDAHV evaluated the previous treatments provided by the MoH of Brazil through SUS since the formulation of the National Plan for Viral Hepatitis in 2002. Past treatments have provided unsatisfactory results; rates of cure were low, and the available therapies did not include a significant share of patients, such as those coinfected with HIV/HCV or presenting advanced liver disease with clinical decompensation [13]. Nearly 50,000 people who have been treated but not cured were waiting for a suitable therapy.

This situation analysis determined that Brazil would need to improve its current response to hepatitis C epidemic over a relatively short-term period. In this context, the MoH of Brazil defined a strategic plan to fulfill three main objectives: 1) improve treatment outcomes by evaluating and incorporating new and effective medications at a sustainable price; 2) elaborate on clinical guidelines suitable for all physicians willing to treat hepatitis C patients; and 3) develop awareness and diagnosis campaigns targeted at the population of interest (people aged 40 years or older).

As the first action, the MoH of Brazil appointed by request a national steering committee on viral hepatitis [14]. This working group comprised representatives of research institutes, higher education institutions, health professionals in charge of the care and treatment of viral hepatitis, and organized civil society. The committee supported the design of the new National Clinical Guidelines, and suggested improvements regarding diagnosis, staging and treatment of viral hepatitis for the universal healthcare system in Brazil.

In order to make evidence-based update of hepatitis C treatment in Brazil, the DDAHV reviewed the evidences from international societies and organizations recommendations [15–17]. The DDAHV also performed a review of the available scientific literature on hepatitis C therapy. The review included Clinical Conference annals, Clinical Trials (I, II, III, IV), Consensus Development Conference, Evaluation Studies, Guidelines, Meta-Analysis, Multicenter Studies, Practice Guidelines, Randomized Controlled Trials, Reviews, and Systematic Reviews, published on the last 5 years. The evidences were classified according to the Grading of Recommendations Assessment, Development and Evaluation (GRADE) system, and submitted to the steering committee for approval.

The bibliographical review submitted for committee evaluation resulted in the selection of three direct-acting antiviral drugs for the national guidelines: daclatasvir, simeprevir and sofosbuvir. Since these drugs had never been tested against each other in clinical trials, the DDAHV ensued by filtering the collection of scientific literature and deconstructing it in cohorts according to the type of study (phase number or real-life); characteristics of the population (presence of coinfection HIV/HCV and severity of disease); HCV genotype (1 to 4); experimented treatment regimen; duration of treatment; and sustained virologic response 12 or 24 weeks after completion of antiviral therapy.

With the objective to implement a sustainable and cost-effective treatment in the public health system, based on estimates of current and future economic impact, the MoH of Brazil performed continuous negotiations with pharmaceutical companies regarding the prices of the proposed medications that would be featured in the new clinical guidelines in substitution of interferon. The pharmaceutical companies were given 6-month period to present suitable prices through an official commercial proposal, with the chance to submit commercial proposals featuring a >90 % discount on international market prices, and to update any reports on the safety and efficacy of their products.

Once the approved commercial proposals were finished and officially registered in October 2014, different segments of the MoH of Brazil evaluated the documents internally. The ANVISA, responsible for the formal approval of new medicines in the Brazilian health system, gave priority for analysis of safety and efficacy of the new medications, evaluated and approved all three drugs by March 2015. Later, the National Committee for Health Technology Incorporation (CONITEC) evaluated and approved the implementation of the new therapies in SUS after the official public consultation.

The intense negotiation with pharmaceutical companies provided Brazil with an unprecedented discount for a high-medium income country, achieving more than 90 % discount over international prices and 20 % over Brazilian reference charts for prices, resulting a final price of under US$ 10,000.00 for 12 weeks of treatment with two oral drugs. Thus, the MoH of Brazil was able to provide treatment to more than 7000 people in the last 2 months of 2015 based on the new guidelines, and it is expected that the MoH will treat at least 38,000 new patients in 2016.

In the meanwhile, the elaboration of the national clinical guidelines focused on eliminating obstacles to access to care and treatment, such as expanding indications for immediate antiviral treatment of hepatitis C to patients with mild to severe liver fibrosis. Patients with coinfection with HIV, chronic kidney disease, malignant neoplasms of lymphoid and blood-forming organs, lichen planus, glomerulonephritis and having undergone solid organ transplant were also granted immediate access to the new antiviral treatment.

The national guidelines were also meant to provide minimum standards of care with routine medical appointments and tests and included less invasive alternatives for the evaluation of liver fibrosis, such as the use of Aspartate Aminotransferase to Platelet Ratio Index (APRI) and Fibrosis-4 (FIB4) scores or elastography, when liver biopsy is not recommended or is simply unavailable. This approach had already been featured in the international recommendations [17], but it had never been fully adapted to the Brazilian context. The purpose of these positive changes were to increase accessibility to hepatitis C treatment, especially for those living in remote areas or locations that lack specialized physicians, and contribute to the cost effectiveness of the health system, decreasing the numbers of unnecessary tests and optimizing care.

In addition, the molecular biology tests routinely performed to treat hepatitis C were reviewed and supplemented with strategic data inputs. This process ensures that the country will have one of the most complete centralized databases in the world, created by the largest real-life cohort for new hepatitis C treatment. Furthermore, it will have relevant data that may help influence current and future pharmaceutical advances.

So at last, the SCTIE and the CONITEC evaluated the proposed clinical guidelines and the economic impact estimates [18] and conveyed all updated recommendations through public consultation to ensure social participation, transparency and accountability in the health system administration.

In October 2015, Brazil began providing interferon-free treatment for hepatitis C using combinations of sofosbuvir + daclatasvir or sofosbuvir + simeprevir. The new clinical guidelines enhances treatment access by including recognized non-invasive technologies as substitutes for liver biopsy when necessary, amplifying the opportunity to achieve the criteria for starting the treatment.

Finally, the DDAHV strengthened public awareness and conducted a new diagnosis campaign in order to debunk the myth that hepatitis C is a chronic incurable disease, and sought to reach the target population through different media. It called for testing those 40 years or older and featured themes regarding past exposure to drugs, unsafe sex, tattoo and medical procedures before 1993, such as surgeries and blood products transfusion, as means to reach the population most at risk of having contracted the hepatitis C virus.

The scientific community and national civil society promptly accepted and supported the successful campaign. Before the first half of 2015, the MoH of Brazil had already distributed the same amount of rapid tests for hepatitis C diagnosis distributed in 2014, and committed to an acquisition of over 8 million rapid tests for the following months.

## Conclusions

Based on the need to refine and to improve the history of committing to the provision of universal access to hepatitis C treatment in Brazil during the last 15 years [4], this remarkable process of evidence-informed policy making–which involved many different actors–changed the Brazilian Clinical Guidelines for Hepatitis C in 2015. Brazil revolutionized the response to hepatitis C epidemic motivated by the nation’s engagement in the Sustainable Development Goals.

The MoH of Brazil managed to fit this response strategy in a 16-month interval by working the momentum of competitive pharmaceutical development scenario to its favor, rising up the bars for epidemiology data analysis, increasing pace in technology evaluation and narrowing communications with scientific community and organized civil society. The standardization of care for hepatitis C also facilitates the flow of treatments, cuts unnecessary costs in laboratory monitoring and facilitates evaluation of incorporated technologies and clinical guidelines. The commitment of different sectors in the MoH of Brazil, the support of all members of the steering committee, the impressive mobilization of civil society, and the flexibility of pharmaceutical companies in the negotiation were crucial for the advancement of this process.

Tackling a public health problem demands full commitment and thorough situation analysis with considerable financial resources for any future policy changes. Such requirements are unlikely to be met simultaneously in every scenario, particularly in viral hepatitis. However, the experience gathered by those countries who were successful in planning and implementation can drastically reduce expenses and invested time.

This comprehensive study of the Brazilian experience allows one to understand that such steps secured means for market scenario development: epidemiology analysis and national guidelines provided numbers of future treatments and effective team work allowed good timing for strategy implementation, thus changing such public health policy changes for viral hepatitis into a win-win condition for any of the parties involved, especially when there is the right to universal access to health. As policy makers, we should do our homework and constantly think out of the box, keeping negotiation channels interesting and open as much as possible.

The national decision was affordable, important and sustainable, and the MoH of Brazil will be able to inspire other developing countries to identify ways to achieve this specific goal of the Sustainable Development Goals by 2030.

To sum it up, there is a moment when the clear understanding of health as human right clashes with the misunderstanding that market based scenarios should be restricted to individual or corporate purchases. Though sometimes rather questionable and far from ideal, mutually beneficial arrangements are quite possible for public health and capitalism. Given the estimates of disease burden, hepatitis C related events case-fatality rates and age of the affected population, it is clear that neither Brazil nor pharmaceutical companies could wait for their own silver bullets any longer and perhaps they should share silver medals instead.

Rest assured, whatever future negotiations hold for Brazil and other countries, nobody will wait much longer for the affordable treatments for hepatitis C.

## Abbreviations

ANVISA: Brazilian Health Surveillance Agency
APRI: Aspartate Aminotransferase to Platelet Ratio Index
CONITEC: National Committee for Health Technology Incorporation
DDAHV: Department of STI, AIDS and Viral Hepatitis
FIB4: Fibrosis-4
GRADE: Grading of Recommendations Assessment, Development and Evaluation
HCV: Hepatitis C virus
MoH: Ministry of Health
SCTIE: Secretariat of Science, Technology and Strategic Inputs
SINAN: Information System on Diseases of Compulsory Declaration
SNT/MS: Brazilian System of Transplants
SUS: Brazilian Unified Health System
SVS: Secretariat of Health Surveillance

## References

[1] Centers for Disease Control and Prevention. Viral Hepatitis–Hepatitis C Information [Internet]. 2016. Available from: http://www.cdc.gov/hepatitis/hcv/hcvfaq.htm. Cited 9 Jun 2016.

[2] Lavanchy D. Evolving epidemiology of hepatitis C virus. Clin Microbiol Infect [Internet]. European Society of Clinical Infectious Diseases; 2011;17(2):107–15. Available from: http://dx.doi.org/10.1111/j.1469-0691.2010.03432.x. 10.1111/j.1469-0691.2010.03432.x 21091831

[3] Lancet T. Hepatitis C: only a step away from elimination? Lancet [Internet]. Elsevier Ltd; 2015;385(9973):1045. Available from: http://dx.doi.org/10.1016/S0140-6736(15)60584-0. 10.1016/S0140-6736(15)60584-0 25797543

[4] Naveira M, Barbosa J, Sereno L, Domanico A, Mesquita F, de Souza LA (2014). 12 years of universal access to hepatitis C treatment: Brazil’s comprehensive response. J Int Assoc Provid AIDS Care.

[5] Dieterich D, Bacon B, Flamm S, Kowdley K, Milligan S, Tsai N, Younossi Z, Lawitz E. Evaluation of sofosbuvir and simeprevir-based regimens in the TRIO network–Academic and community treatment of a real-world, heterogeneous population [Internet]. 2014. Available from: http://www.natap.org/2014/AASLD/AASLD_09.htm. Accessed 9 June 2016.

[6] Afdhal N, Everson GT, Calleja JL, McCaughan G, Symonds WT, Denning JM, et al. Sofosbuvir and ribavirin for the treatment of chronic HCV with cirrhosis and portal hypertension with and without decompensation: early virologic response and safety. J Hepatol [Internet]. Elsevier B.V. and European Association for the Study of the Liver; 2014;60(Supplement 1):S28. Available from: http://dx.doi.org/10.1016/S0168-8278(14)60070-2.

[7] Jennings CL, Sherman KE (2012). Hepatitis c and HIV co-infection: New drugs in practice and in the pipeline. Curr HIV/AIDS Rep.

[8] Hill A, Khoo S, Fortunak J, Simmons B, Ford N (2014). Minimum costs for producing hepatitis C direct-acting antivirals for use in large-scale treatment access programs in developing countries. Clin Infect Dis.

[9] Amaku M, Burattini MN, Coutinho FAB, Lopez LF, Mesquita F, Naveira MCM, et al. Estimating the Size of the HCV Infection Prevalence: A Modeling Approach Using the Incidence of Cases Reported to an Official Notification System. Bull Math Biol [Internet]. Springer US; 2016; Available from: http://link.springer.com/10.1007/s11538-016-0170-4. 10.1007/s11538-016-0170-4 27160282

[10] Brasil. Ministério da Saúde. Sistema de Informação de Agravos de Notificação [Internet]. 2016. Available from: http://portalsinan.saude.gov.br/. Accessed 9 June 2016.

[11] Coutinho FAB. Modeling and restoring a defective data base on hepatitis C [Internet]. 2015. Available from: http://hepatitis.omicsgroup.com/abstract/2015/modeling-and-restoring-a-defective-data-base-on-hepatitis-c. Accessed 9 June 2016.

[12] Brasil. Ministério da Saúde. Boletim Epidemiológico Hepatites Virais. 2015;15. Available from: http://www.aids.gov.br/sites/default/files/anexos/publicacao/2015/58210/_p_boletim_hepatites_final_web_pdf_p__16377.pdf. Accessed 9 June 2016.

[13] Brasil. Ministério da Saúde. Simeprevir, sofosbuvir e daclatasvir no tratamento da hepatite crônica tipo C e coinfecções. 2015;112. Available from: http://conitec.gov.br/images/Consultas/Relatorios/2015/Relatorio_Antivirais_HCV_CP.pdf. Accessed 9 June 2016.

[14] Brasil. Ministério da Saúde. Portaria n^o^ 4, de 6 de fevereiro de 2014. Diário Of da União; 2014. p. 55.

[15] Chung RT, Davis GL, Jensen DM, Masur H, Saag MS, Thomas DL (2015). Hepatitis C guidance: AASLD-IDSA recommendations for testing, managing, and treating adults infected with hepatitis C virus. Hepatology.

[16] Pawlotsky J-M, Al E (2015). EASL Recommendations on Treatment of Hepatitis C 2014. Hepatology.

[17] World Health Organization. Guidelines for the screening, care and treatment of persons with hepatitis c infection. Guidelines. 2014;(April):124, ISBN 978 92 4 154875 5. 25535634

[18] Brasil. Ministério da Saúde. Protocolo Clínico e Diretrizes Terapêuticas para Hepatite C e Coinfecções. 2015. Available from: http://conitec.gov.br/images/Consultas/Relatorios/2015/Relatorio_PCDT-HepatiteC-CP.pdf. Accessed 9 June 2016.

